# Autophagy Plays a Role in the CUL4A-Related Poor Prognosis of Intrahepatic Cholangiocarcinoma

**DOI:** 10.3389/pore.2021.602714

**Published:** 2021-02-23

**Authors:** Shao-Wen Weng, Ting-Ting Liu, Hock-Liew Eng, Huey-Ling You, Wan-Ting Huang

**Affiliations:** ^1^ Department of Internal Medicine, Kaohsiung Chang Gung Memorial Hospital and Chang Gung University College of Medicine, Kaohsiung, Taiwan; ^2^ Department of Pathology, Kaohsiung Chang Gung Memorial Hospital and Chang Gung University College of Medicine, Kaohsiung, Taiwan; ^3^ Department of Medical Laboratory Science, I-Shou University, Kaohsiung, Taiwan; ^4^ Department of Laboratory Medicine, Kaohsiung Chang Gung Memorial Hospital and Chang Gung University College of Medicine, Kaohsiung, Taiwan; ^5^ Department of Medical Laboratory Sciences and Biotechnology, Fooyin University, Kaohsiung, Taiwan

**Keywords:** autophagy, CUL4A, intrahepatic cholangiocarcinoma, prognosis, LC3II

## Abstract

CUL4A regulate the termination of autophagy in a physical process. However, the relationship between CUL4A and autophagy in cancer is unclear. We retrospectively investigated 99 intrahepatic cholangiocarcinoma (iCCA) cases. Whole sections were used for immunohistochemical analysis for p62, and LC3B expression. Q-score was defined as the sum of the labeling intensity and proportion. The cut-off point for immunoreactivity was set. CUL4A was overexpressed in cell lines and autophagy reflux was compared after manipulation. The iCCA cases with CUL4A overexpression had significantly higher prevalence of intact activated autophagy (42.4 vs. 15.2%; *p* = 0.003), which was significantly associated with advance tumor stage (34.1% vs. 15.4%; *p* = 0.032), less extensive necrosis (8.3 vs. 49.3%; *p* < 0.001), and shortened disease-free survival (mean, 19.6 vs. 65.5 months, *p* = 0.015). *In vitro*, iCCA cells with CUL4A overexpression significantly increased LC3II level as compared to the cells under basal condition. Although both cell types showed intact autophagy with increased LC3II expression after bafilomycin A1 treatment, the accumulation of LC3II was higher in CUL4A-overexpressing cells. CUL4A overexpression increased the proliferation of cells as compared with control cells. After treatment with bafilomycin A1, proliferation was inhibited in both cell types, but the effects were more prominent in the cells overexpressing CUL4A. CUL4A promotes autophagy, and exhibits significantly higher autophagic flux which affects the proliferation of iCCA cells; these effects correlated with advance tumor stage and poor prognosis. Thus, targeting autophagy may be potentially therapeutic in iCCA.

**GRAPHICAL ABSTRACT:**
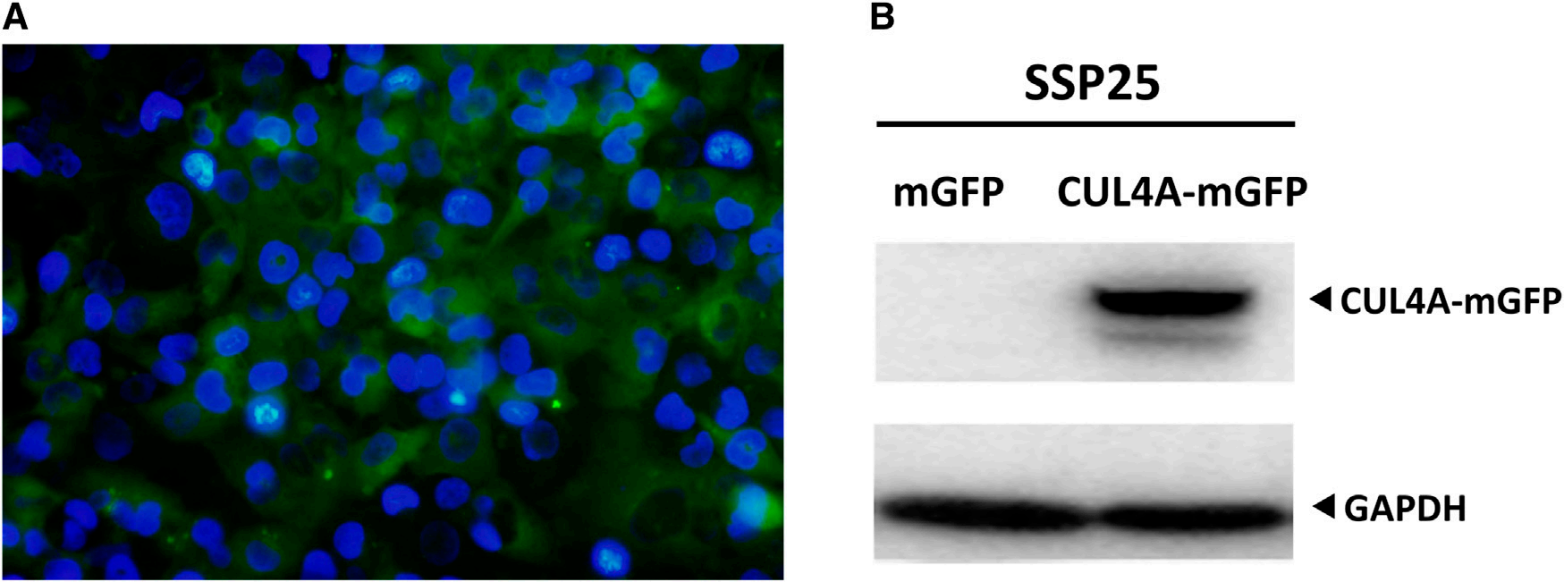
Overexpression of CUL4A in iCCA cells. **(A)** SSP25 cells were co-transfected with pLenti-C-mGFP-CUL4A plasmid along with lentiviral packaging mix for lentiviral production. The expression of the reporter gene in the lentivirus was observed by immunofluorescence staining for green fluorescent protein (GFP) along with DAPI staining. Percentage of cells expressing CUL4A-mGFP was normalized to DAPI-stained nuclei (blue). The image was obtained at a magnification of 200x. **(B)** Total cell lysates were analyzed for CUL4A protein levels by western blotting. GAPDH was used as the loading control.

## Introduction

CUL4A is a member of the cullin family of proteins and comprises a multifunctional ubiquitin-protein ligase E3 complex. This cullin-RING ubiquitin ligase (CRL) mediates the process of ubiquitylation or ubiquitination through a cascade of enzymatic reactions involving E1, E2, and E3 enzymes, which play a vital role in the regulation of cellular physiology [1]. CUL4A is physically associated with the mouse double minute 2 homolog (MDM2)-mediated proteolysis of p53 through the ubiquitin-proteasome pathway [2]. Any deregulation in *CUL4A* copy number and gene expression may result in an extreme effect on cells, eventually leading to tumorigenesis.

*CUL4A* was first found to be amplified and overexpressed in primary breast cancers in 1998 [3], and was eventually reported in hepatocellular carcinomas [4], malignant pleural methotheliomas [5], and prostate cancers [6]. Overexpression of *CUL4A* is associated with tumor proliferation, progression, and metastasis [7, 8]. Wang et al. found that CUL4A overexpression would increase epithelial-mesenchymal transition (EMT) and promote the metastatic capacity of breast cancer cells [8]. The role of CUL4A in intrahepatic cholangiocarcinoma (iCCA) has been rarely explored. Our previous findings suggest the importance of CUL4A in tumor progression [9, 10].

Autophagy maintains cellular homeostasis by degrading harmful or unnecessary intracellular components. The mechanism underlying the transient yet rapid induction of autophagy response is, however, unclear. The role of autophagy in cancer is controversial, but autophagy is thought to preserve organelle function, prevent the toxic buildup of cellular waste products, and provide substrates to sustain metabolism under starvation. While autophagy suppresses tumorigenesis in some cases, it may facilitate tumorigenesis in others [11]. Autophagy was initially thought to be a tumor suppression mechanism, as suggested by early studies reporting the monoallelic loss of the essential autophagy gene *ATG6*/*BECN1* in 40–75% of human prostate, breast, and ovarian cancers [12]. While cancer cells also rely on autophagy; in many cases, they are more autophagy dependent than normal cells and tissues. This observation is likely attributed to the increase in metabolic and biosynthesis demands imposed by deregulated proliferation [13]. Some mechanisms support the role of autophagy in promoting cancer growth by limiting stress responses and supporting metabolism and survival [12].

Considering the association between CUL4A and autophagy, a previous report revealed that CUL4A regulates the termination of autophagy in a physical process [14]. However, the relationship between CUL4A and autophagy in cancer is still unclear. In the present study, we evaluated the effects of *CUL4A* overexpression on autophagy to elucidate the possible mechanisms underlying iCCA tumor progression.

## Materials and Methods

### Patient Samples

We selected 99 cases with or without CUL4A expression in our previous study [10]. The inclusion criteria included presence of viable and potentially adequate tumors for immunostaining of whole sections. This study was approved by the Institutional Review Board of Chang Gung Memorial Hospital. The clinical staging was determined according to the American Joint Committee on Cancer (AJCC) 7th edition staging system.

### Immunohistochemical Analysis

Immunostaining of whole sections was performed by deparaffinizing the sections in xylene and subsequent hydration in a graded series of alcohol. Endogenous peroxidase was blocked with 3% hydrogen peroxide. IHC study was performed with BioGenex system using an automated i6000 immunostainer (BioGenex Corp.) and primary antibodies to microtubule-associated protein 1 light chain 3 beta (LC3B) (clone AA 77–106, 1:400, Antibodies-Online) and p62/sequestosome-1 (clone sc-28359, 1:50, Santa Cruz Biotechnology Inc., CA, United States). Slides were evaluated by two pathologists (WTH and TTL), blinded to clinicopathological data.

We referred to the scoring methods reported by Schläfli et al. [15] and Falasca et al. [16]. Labeling intensity was scored from 0 to 3 corresponding to negative, weak, moderate, and strong staining, respectively. Staining proportion was scored as 0 (<5%), 1 (5–25%), 2 (26–50%), 3 (51–75%), or 4 (76–100%). Q-score was defined as the sum of the labeling intensity and proportion. The cut-off point for immunoreactivity was settled as follows: p62, Q-score >2 and LC3II, Q-score >2.

### Cell Lines and Stable Transfection

The SSP 25 iCCA cell line was purchased from the Riken BRC Cell Bank (Koyadai, Japan). Cells were cultured in Roswell Park Memorial Institute (RPMI) medium (Gibco-BRL, CA, United States). The lentivirus vectors carrying *CUL4A* were purchased from OriGene (OriGene, MD, United States). HEK293NT (human embryonic kidney, ATCC CRL-1573) cells were co-transfected with control vector or pLenti-C-mGFP-CUL4A plasmid along with lentiviral packaging mix (OriGene, MD, United States) for lentiviral production. The culture medium was collected at 24 and 48 h after transfection. The iCCA cells were seeded in six-well plates for 12 h before transduction. After infection, the expression of the reporter gene in the lentivirus was evaluated from green fluorescent protein (GFP) signal. The ratio of lentivirus-infected cells was observed under an inverted fluorescence microscope (Olympus, Tokyo, Japan). Puromycin was added to select GFP-positive cells. Single foci were isolated and subsequently expanded into permanent monoclonal cell lines. Total cell lysates were analyzed by western blotting.

### Western Blot Analysis

Western blotting was performed with a sodium dodecyl sulfate-polyacrylamide gel electrophoresis system as previously described [9, 10]. Immunoblotting was performed following incubation of samples with CUL4A (clone EPR3198, 1:10000, Abcam, Cambridge, MA), LC3B (catalog no. NB100–2220, polyclonal, 1:2000, Novus Biologicals, Littleton, CO), and glyceraldehyde 3-phosphate dehydrogenase (GAPDH; clone 6C5, 1:10000, Millipore, Burlington, MA) antibodies at 25°C for 2 h. The blots were washed and incubated with a 1:2000 dilution of horseradish peroxidase (HRP)-conjugated secondary antibody (Jackson, West Grove, PA, United States), followed by three washes with TBST (Tris-buffered saline + Tween). Enhanced chemiluminescent HRP substrate (Pierce, Rockford, IL, United States) was used for detection according to manufacturer’s description.

### Autophagic Flux Study to Measure Autophagic Clearance

The iCCA cells were treated in the presence or absence of 100 nm bafilomycin A1 (catalog no. tlrl-baf1, InvivoGen San Diego, CA) and examined for LC3 expression at 0, 2, 4, and 6 h. LC3 expression was evaluated using western blotting with GAPDH used as a loading control. Values indicative of autophagic flux were quantitatively determined from the expression difference in LC3II between cells treated with and without bafilomycin A1.

### Effect of Autophagy on Cell Growth

The iCCA cells (2.0 × 10^3^ cells/well) were seeded into 96-well culture plates and incubated for three different time periods (24, 48, and 72 h). XTT (tetrazolium hydroxide salt) reagent (Roche, Basel, Switzerland) was added and the cells were incubated for additional 4 h. The absorbance of the resulting solution was measured at 570 nm wavelength with a reference wavelength of 650 nm using Sunrise microplate reader (Tecan, Männedorf, Switzerland). Cells were treated with varying concentrations of bafilomycin A1 (0, 1, 2.5, 5, and 10 nm) for indicated time periods and their viability was determined with the XTT assay, as previously described [10]. Each experiment was carried out in triplicates and separately performed at least thrice.

### Statistical Analysis

The chi-square test, Fisher’s exact test, and *t*-test were used to compare the data between the two groups. Overall survival (OS) was calculated from the date of diagnosis to that of death as a result of all causes. Disease-free survival (DFS) was computed from the time of surgery to time of recurrence in the liver or distant metastasis. The Kaplan Meier method was used for univariate survival analysis, and the difference between survival curves was tested by a log-rank test. Cox regression analysis was performed to analyze the relative prognostic importance. All statistical analyses were performed using Statistical Product and Service Solutions (SPSS) for Windows 17.0 software (SPSS Inc. Chicago, IL). A value of *p* < 0.05 for two-tailed tests was considered statistically significant.

## Results

### Clinicopathologic Features

**FIGURE 1:**
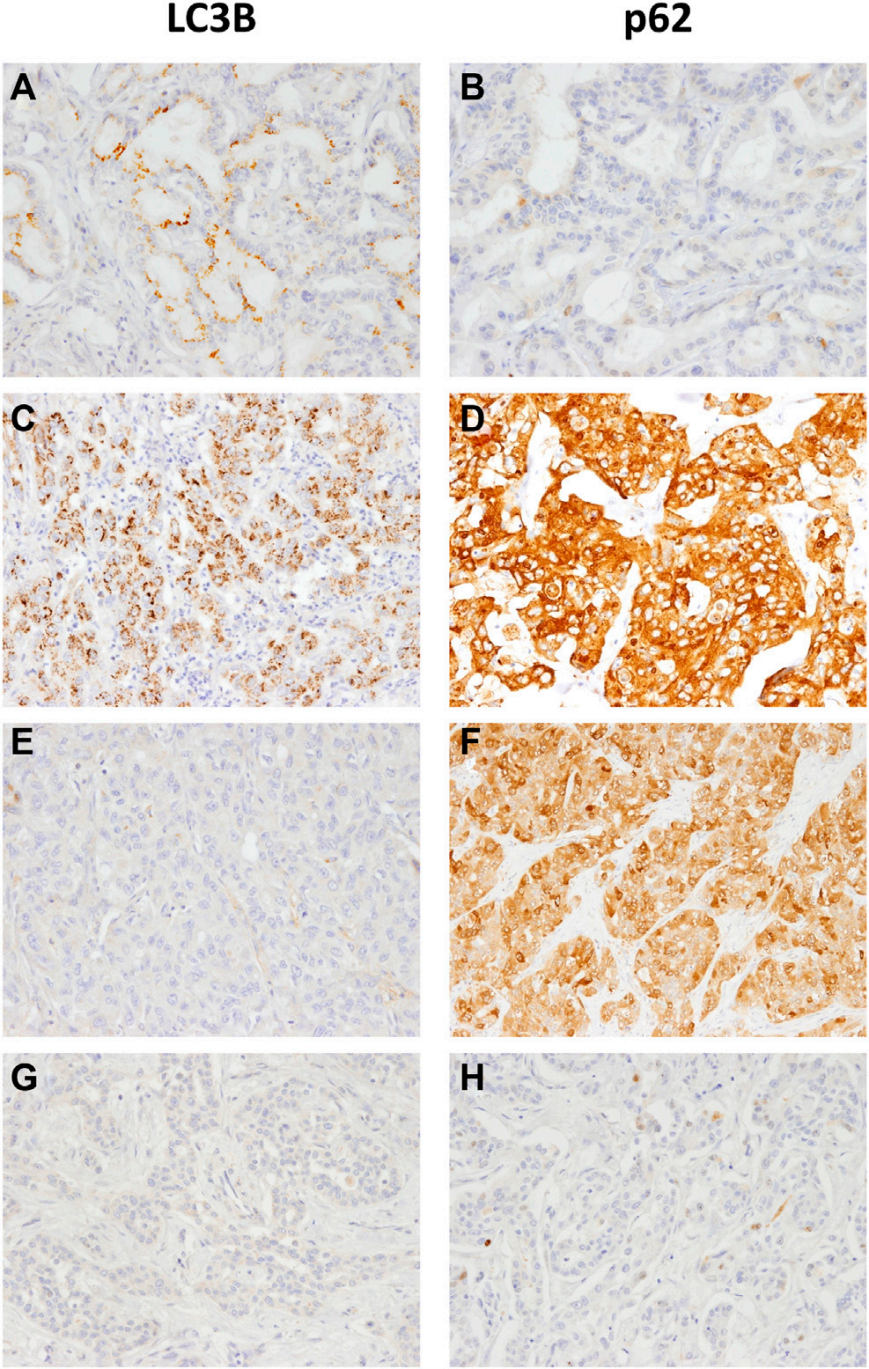
Representative LC3B and p62 immunohistochemical staining in intrahepatic cholangiocarcinoma samples **(A, B)** LC3B dot-like staining and negative p62 indicates intact activated autophagy **(C, D)** LC3B dot-like staining and strong p62 cytoplasmic **(E, F)** no LC3B staining and positive p62 expression or **(G and H)** negative staining for both LC3B and p62 indicate the non-intact activated autophagy (magnification, 200×).

We performed IHC staining for whole tissue sections from 99 iCCA samples. We integrated autophagic markers with CUL4A expression. Two autophagy phenotypes, intact activated type and non-intact type, were clarified (Figure 1). The phenotypes were defined according to the expression of LC3B and p62 as follows [17, 18]: (1) Intact activated type: LC3B+/p62− (high LC3B and low p62) and (2) non-intact type: LC3B+/p62+ (high LC3B and high p62) indicative of autophagy activation, which was impaired at later steps during the process; LC3B−/p62+ (low LC3B and high p62) representing a basal level of autophagy which was impaired at later steps of process; and LC3B−/p62− (low LC3B and low p62) mimicked the basal level of autophagy.

**FIGURE 2:**
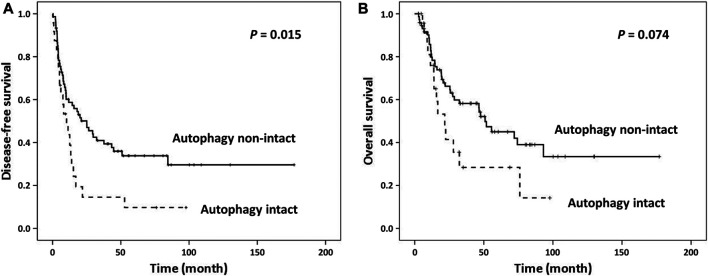
Kaplan Meier survival curves of patients categorized by autophagy. **(A)** Intact activated autophagy was significantly associated with shortened disease-free survival. **(B)** Marginal significantly shortened overall survival was observed between groups.

Samples with CUL4A overexpression had significantly higher prevalence of intact activated autophagy (42.4 vs. 15.2%; *p* = 0.003). Furthermore, intact activated autophagy was significantly associated with advance tumor stage (34.1 vs. 15.4%; *p* = 0.032) and less extensive necrosis (8.3 vs. 49.3%; *p* < 0.001) (Table 1). DFS of cancer patients with intact activated autophagy was significantly shorter (mean, 19.6 vs. 65.5 months; *p* = 0.015; Figure 2A). Analysis of OS revealed a decreasing trend in the survival time for patients with intact activated autophagy (mean, 36.7 vs. 81.7 months; *p* = 0.074; Figure 2B). Multivariate Cox proportional hazard regression analysis was used to derive the risk estimates related to DFS for clinicopathologic factors. After correction for tumor size, tumor stage, and resection margin, we found that intact activated autophagy was related to shortened DFS with a hazard ratio of 1.75 (*p* = 0.078) (Table 2).

**TABLE 1:** Clinicopathological characteristics and association with autophagy of patients with intrahepatic cholangiocarcinoma.

Parameters	No. of patients	Intact activated autophagy		*p* Value
		Yes	No	
Age, year				
≤60	52	10	42	0.221
>60	47	14	33	
Gender				
Male	56	13	43	0.785
Female	43	11	32	
Tumor size				
≤5 cm	47	13	34	0.215
>5 cm	42	7	35	
Necrosis				
≤10%	60	22	38	<0.001^*^
>10%	39	2	37	
VI				
No	58	17	41	0.162
Yes	41	7	34	
NI				
No	61	12	49	0.179
Yes	38	12	26	
H Grade				
I + II	83	23	60	0.067
III	16	1	15	
Stage				
I + II	52	8	44	0.032^*^
III + IV	44	15	29	
CUL4A overexpression				
No	66	10	56	0.003^*^
Yes	33	14	19	

NS, not signtificant; N, number; VI, vascular invasion; NI, neural invasion; H, histology

^*^ Statistically significan

**TABLE 2:** Independent predictive factors of disease-free survival from multivariate analysis.

Variable	Hazard ratio	95% CI	*P*
Tumor size ≤5 vs. > 5 cm	2.66	1.54 to 4.60	<0.001
Resection margin ≤1 vs. > 1 cm	1.57	0.80 to 3.08	0.189
Stage I andII vs. III and IV	2.15	1.23 to 3.76	0.008
Intact vs. non-intact activated autophagy	1.75	0.94 to 3.24	0.078

#### CUL4A Transfection in iCCA Cells Increased Autophagic Flux, Cell Proliferation and Susceptibility to Bafilomycin A1.

To understand the interplay between CUL4A and autophagy, we established iCCA cells stably overexpressing CUL4A to study autophagic clearance (Supplementary Material). Bafilomycin A1 was a specific inhibitor of vacuolar-type H + -ATPase that blocked autophagosome-lysosome fusion and caused accumulation of LC3II. Values indicative of autophagic flux was quantitatively determined by expression difference of LC3II amount between cells treated with and without bafilomycin A1.

**FIGURE 3:**
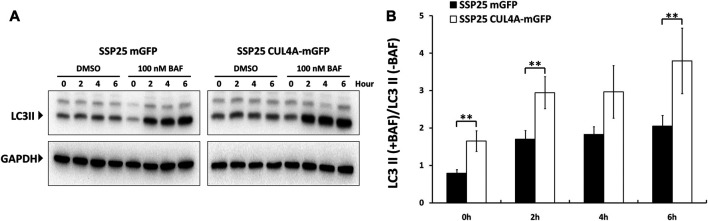
Comparisons of autophagic flux between iCCA cells with or without CUL4A overexpression. Cells with CUL4A overexpression showed a much greater accumulation of LC3II in CUL4A overexpression cells after bafilomycin A1 treatment. **(A)** The iCCA cells were treated with the presence or absence of 100 nm bafilomycin A1 and examined LC3 expression in the course of the indicated time point. The levels of LC3II expression are examined by Immunoblotting followed by quantitative analysis using Image**J**. Data are normalized to GAPDH level. **(B)** Values indicative of autophagic flux are quantitatively determined by expression difference of LC3II amount between cells treated with DMSO and cells treated with bafilomycin A1. The scale represents ratio of LC3II following bafilomycin A1 treatment/untreated. Data represent means ± standard deviation from three independent experiments. **, *p* < 0.01.

Cells with CUL4A overexpression showed a significant increase in LC3II level as compared with the cells cultured under basal condition. Although both cell types showed intact autophagy with increased LC3II expression after bafilomycin A1 treatment, the accumulation of LC3II was much higher in CUL4A-overexpressing cells. These differences in the level of LC3II in the presence and absence of bafilomycin A1 revealed that CUL4A promotes autophagy and is associated with significantly higher autophagic flux (Figure 3).

**FIGURE 4:**
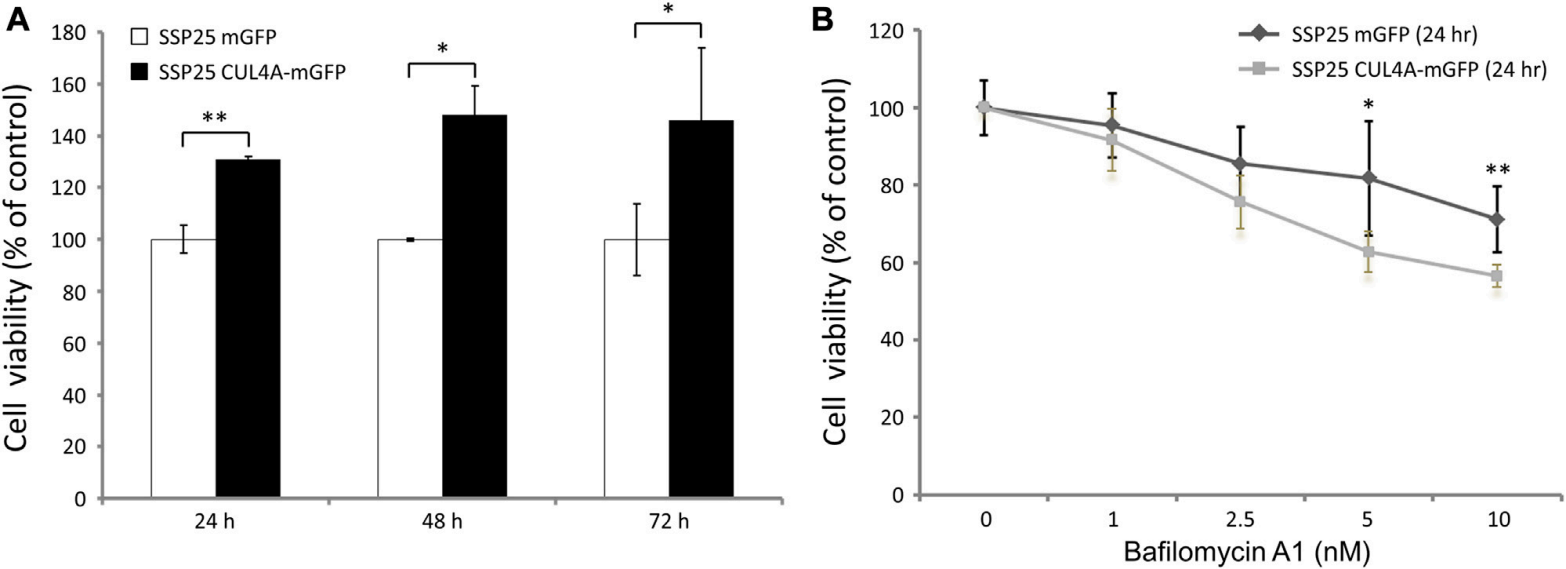
Effects of bafilomycin A1 on iCCA cells. **(A)** Cell viability was assessed with the XTT assay at 24, 48, and 72 h. The results are presented as percentage viability relative to cells from the vehicle control group. **(B)** We treated iCCA cells with 0, 1, 2.5, 5, and 10 nm of bafilomycin A1 for indicated time periods. The results of iCCA cells treated with varying concentrations of bafilomycin A1 for 24 h are presented as percentage viability relative to untreated control cells. Data represent means ± standard deviation for triplicates of each experiment from three independent determinations. *, *p* < 0.05; **, *p* < 0.01.

The cells overexpressing CUL4A showed an increase in proliferation as compared with the vehicle control cells (Figure 4A). After treatment with bafilomycin A1, cell proliferation was inhibited in both groups, but the decline was significantly dominant in the cells overexpressing CUL4A incubated for 24 h (Figure 4B).

## Discussion

The role for autophagy in cancer is still controversial. Autophagy preserves organelle function, prevents the toxic buildup of cellular waste products, and provides substrates to sustain metabolism under starvation. While cancer cells also rely on autophagy; in many cases, they are more autophagy dependent than normal cells and tissues [13]. Our previous study showed that patients with tumors overexpressing CUL4A showed significantly shortened DFS [10]. In the present study, we elucidated the clinical effects of CUL4A in iCCA that are probably mediated through intact activated autophagy, which affects the proliferation of iCCA cells; these effects correlated with advance tumor stage and poor prognosis.

Some studies suggest that autophagy promotes cancer by limiting stress responses and supporting metabolism and survival [12]. Autophagy has been shown to be involved in the modulation of the migratory capacity of cancer cells [11], and emerging evidences have revealed the link between autophagy and EMT [19, 20]. CUL4A was shown to regulate the termination of autophagy in a physical process [14] and our previous study demonstrated the positive correlation between CUL4A expression and tumor invasion and poor prognosis of iCCA [10]. Thus, it is important to elucidate the link between CUL4A and autophagy in iCCA. In the present study, in contrast to the physical process, we found that CUL4A promotes intact autophagy in iCCA, thereby supporting the relationship with aggressive iCCA. Autophagy was initially thought to be a tumor suppression mechanism, as proposed by the studies demonstrating the monoallelic loss of the essential autophagy gene *ATG6/BECN1* in 40–75% of human prostate, breast, and ovarian cancers [12]. In certain cell-based assays, autophagy suppression promoted cancer cell growth, and *Becn1* heterozygous mutant mice were prone to liver and lung tumors and lymphomas with long latency [21, 22]. Together, autophagy may play dual effects under different genetic background or in different cancers. Further research is necessary to elucidate it.

p62 is a ubiquitin-dependent adaptor and serves as a cargo receptor for selective autophagy of ubiquitinated targets. Together with LC3II, p62 serves as the surrogate marker commonly used to identify any association between autophagy and subtypes of cancers in primary human tumor samples. Niklaus et al. reported the combination of the expression pattern of high LC3II and low p62 levels as an indication of intact activated autophagy and its association with worst prognosis in colon cancer [18]. The result of single staining analysis of LC3II and p62 showed the positive correlation between autophagy and metastases of breast cancer, melanoma, oral squamous cell carcinoma, and hepatocellular carcinoma [17, 23–25]. However, impaired autophagy characterized with high p62 cytoplasmic expression regardless of LC3B expression correlated with unfavorable outcomes in oral squamous cell carcinoma [17]. These *ex vivo* studies suggest that autophagy may regulate cancer progression in a context-dependent manner through different regulatory pathways [26].

*CUL4A* functions as an oncogene in several cancers. Overexpression of CUL4A is associated with tumor proliferation, progression, and metastasis. Studies have clarified the role of microRNAs (miRs) in the regulation of CULA expression for EMT, and the relationship between CUL4A expression silencing and chemosensitivity. MiR-377 and miR-494 were reported as negative regulators of CUL4A expression in ovarian cancer, and significantly reduced the migratory ability of cancer cells [27, 28]. The knockdown of *CUL4A* expression was shown to increase the chemosensitivity of lung cancer cells [29, 30]. Taken together, CUL4A is an adverse prognostic factor and may serve as a potential marker for prognosis and therapy. In the present study, we found that CUL4A-overexpressing iCCA cells had intact activated autophagy and higher autophagic flux. Hence, targeting autophagy may serve as a potential therapeutic strategy against CULA4-overexpressing iCCA. Chloroquine and hydroxychloroquine that inhibit autophagosome-lysosome fusion are the only autophagy inhibitors approved by the FDA for clinical trials [31]. The inhibitors for small molecule modulators of epigenetics are other promising agents targeting autophagy. Ellagic acid is an inhibitor of CARM1-mediated H3R17 methylation with a potential therapeutic value for autophagy inhibition [32]. S-adenosyl-L-methionine competitive inhibitors block the negative epigenetic regulation of autophagy by EZH2 trimehtylating H3K27 to induce autophagic cell death [33]. These studies may shed a new light on therapy of iCCA.

In conclusion, our data demonstrate that CUL4A promotes autophagy and induces significantly higher autophagic flux in iCCA. The results of our current and previous studies show that CUL4A exhibits a positive correlation with poor prognosis of iCCA, probably through intact activated autophagy, and that targeting autophagy may be a potential therapeutic approach for iCCA treatment.

## Data Availability

The original contributions presented in the study are included in the Supplementary Material, further inquiries can be directed to the corresponding author.
