# Supplementary material for: Autophagy Plays a Role in the CUL4A-Related Poor Prognosis of Intrahepatic Cholangiocarcinoma
Source: Pathol Oncol Res. 2021 Feb 23;27:602714. doi: 10.3389/pore.2021.602714 (PMC8262180; doi:10.3389/pore.2021.602714)
Supplement: Supplementary file 1 [file Table1.DOCX]

**
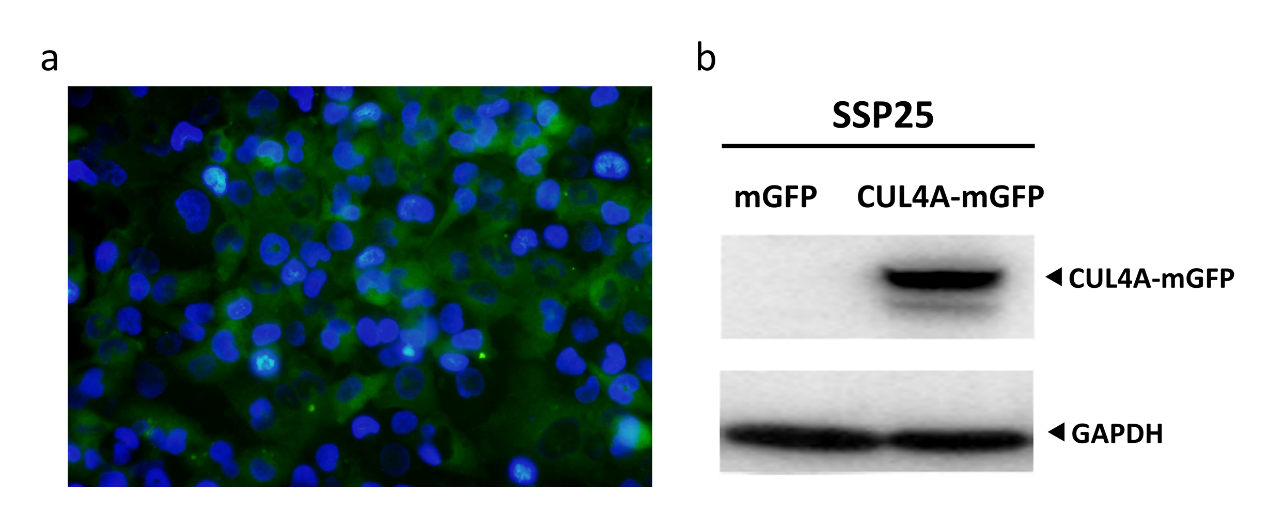
** Overexpression of CUL4A in iCCA cells. (**a**) SSP25 cells were co-transfected with pLenti-C-mGFP-CUL4A plasmid along with lentiviral packaging mix for lentiviral production. The expression of the reporter gene in the lentivirus was observed by immunofluorescence staining for green fluorescent protein (GFP) along with DAPI staining. Percentage of cells expressing CUL4A-mGFP was normalized to DAPI-stained nuclei (blue). The image was obtained at a magnification of 200×. (**b**) Total cell lysates were analyzed for CUL4A protein levels by western blotting. GAPDH was used as the loading control.
